# Supplementary material for: FERN – a Java framework for stochastic simulation and evaluation of reaction networks
Source: BMC Bioinformatics. 2008 Aug 29;9:356. doi: 10.1186/1471-2105-9-356 (PMC2553347; doi:10.1186/1471-2105-9-356)
Supplement: Additional file 1 — FERN distribution, Version 1.3. This archive contains the FERN source code and binaries as well as documentation and example models in FernML and SBML. [file 1471-2105-9-356-S1.zip › fern/doc/javadoc/overview-frame.html]

Overview List


All Classes

Packages
  
fern
  
fern.analysis
  
fern.benchmark
  
fern.cellDesigner
  
fern.cellDesigner.ui
  
fern.cytoscape
  
fern.cytoscape.ui
  
fern.example
  
fern.network
  
fern.network.creation
  
fern.network.fernml
  
fern.network.modification
  
fern.network.sbml
  
fern.simulation
  
fern.simulation.algorithm
  
fern.simulation.controller
  
fern.simulation.observer
  
fern.tools
  
fern.tools.functions
  
fern.tools.gnuplot
